# Implementation supports to promote fidelity within a flexible, presentation-responsive MHPSS intervention model: A case study of Baby Friendly Spaces in Cox’s Bazar, Bangladesh

**DOI:** 10.1192/j.eurpsy.2024.1500

**Published:** 2024-08-27

**Authors:** K. Le Roch, K. S. Rahaman, P. Bubendorff, L. Clouin, S. M. Murray, A. J. Nguyen

**Affiliations:** ^1^Mental Health and Psychosocial Support, Action contre la Faim, Montreuil, France; ^2^Mental Health and Psychosocial Support, Action contre la Faim, Cox’s Bazar, Bangladesh; ^3^MHPSS Independent expert, Nantes, France; ^4^Mental Health, Johns Hopkins Bloomberg School of Public Health, Baltimore; ^5^School of Education and Human Development, University of Virginia, Charlottesville, United States

## Abstract

**Introduction:**

As evidence has converged on the feasibility and effectiveness of focused, non-specialized, manualized interventions for treating mental distress in humanitarian settings, challenges persist in how to promote implementation fidelity and rigorously evaluate interventions designed to be more preventive or promotive in addressing risk and protective factors for poor mental health. One such intervention, Baby Friendly Spaces (BFS), is a psychosocial support program implemented for Rohingya mothers and their malnourished children living in refugee camps of Cox’s Bazar, Bangladesh. That follows a place-based intervention model in which various activities may be offered either individually or in groups with no specified sequence.

**Objectives:**

This presentation describes the process of establishing standards for implementing optimal mental health and psychosocial support (MHPSS) interventions, training BFS workers, and building monitoring and supervision systems to promote implementation fidelity within this flexible support program.

**Methods:**

As BFS services were already being offered as part of Action Against Hunger programming, we first conducted an audit of current services, determining that there was limited current standardization or support for implementation. Therefore, a manualized protocol was designed and covered the program curricula and self-care using didactic and practice-based learning. A series of online training sessions were conducted for 13 psychosocial workers and psychologists at centers delivering the enhanced intervention. Following the training, a baseline evaluation of attitudes, confidence, and knowledge for delivering BFS services was administered. We also collaboratively designed a systematic supervision process to meet the staff’s needs with a focus on capacity building and self-care.

**Results:**

Following the initial training, BFS workers receiving the re-training showed similar levels of knowledge, but greater confidence (p=0.01) than MHPSS workers proceeding as usual. Participants reported that the training was useful for their field of work and for improving the quality of their work, and acknowledged they would be able to integrate the new learnings into their work and daily life. The follow-up with the supervision process confirmed their capacity to deliver the services and highlighted the need for workspace improvements, the lack of continuous motivation, their ability to identify specific issues for which they requested additional trainings.

**Conclusions:**

There is a particular need for careful attention to implementation supports and supervision when offering flexible, place-based mental health and psychosocial support interventions. In that process, ensuring a continuity between the training and the supervision is essential for the quality of both the program and the research project.

**Disclosure of Interest:**

None Declared

